# Postoperative hemicrania continua-like headache - a case series

**DOI:** 10.1186/s10194-015-0526-4

**Published:** 2015-05-06

**Authors:** Andreas R Gantenbein, Hakan Sarikaya, Franz Riederer, Peter J Goadsby

**Affiliations:** Neurorehabilitation, RehaClinic Bad Zurzach, Bad Zurzach, Switzerland; University of Zurich, Zurich, Switzerland; Neurological Practice, Reinach and Department of Neurology, University Hospital Inselspital, Bern, Switzerland; Karl Landsteiner Institute for Clinical Epilepsy Research and Cognitive Neurology, Neurological Center Rosenhuegel, Vienna, Austria; NIHR-Wellcome Trust Clinical Research Facility, King’s College London, UK and Headache Group-Department of Neurology, University of California, San Francisco, CA USA

**Keywords:** Hemicrania contiuna, Neurosurgery, Craniotomy, Postoperative, Indomethacin, TAC

## Abstract

**Background:**

Hemicrania continua (HC) is a rare chronic headache disorder, typically accompanied by cranial autonomic features and responding to therapeutic doses of indomethacin. The pathophysiology of hemicrania continua is not fully understood.

**Findings:**

We report a series of three patients who developed a continuous hemicranial headache after cranial surgery. Each case presented a similar phenotype of continuous half-sided headache, cranial autonomic symptoms with exacerbations (2/3), and a response to indomethacin.

**Conclusion:**

The biology of hemicrania continua may be activated post-craniotomy just as can be seen with other primary headache disorders.

## Introduction

Hemicrania continua (HC) is a rare primary headache disorder described initially by Sjaastad and Spierings in 1984 [1]. The continuous half-sided headache is typically completely resolved by indomethacin. Exacerbations of the pain are accompanied by unilateral cranial autonomic symptoms including, conjunctival injection, lacrimation, ptosis, nasal congestion or rhinorrhoea. Agitation or restlessness have been described [2]. In the 2^nd^ edition of the Internal Classification of Headache Disorders (ICHD-II), HC was not listed among the trigeminal-autonomic cephalalgias. However, as similar pathophysiological mechanisms have been suggested [3], it has been included among the trigeminal autonomic cephalalgias in the 3^rd^ edition beta version [4]. An involvement of the trigeminal-autonomic reflex, either centrally or peripherally, seems to be important.

Post-operative headache (POH) or post-craniotomy headache (PCH) is poorly understood. Nerve entrapment, meningeal inflammation and dural adhesions are some of the discussed mechanisms. The occurrence of headache after cranial surgery is dependent on the neurosurgical approach, with, in particular, posterior fossa procedures being most likely to be associated with POH [5]. PCH was included in the ICHD-II, but the characteristics remain unspecific. It could be part of a broader spectrum of post-traumatic headache, where surgery is the trauma.

Ashkenazi et al. reported an HC-like headache as presenting symptom of a dissection of the internal carotid artery [6]. In 1999 Lay and Newman reported four patients whose onset of indomethacin-responsive hemicrania continua was temporally linked to head trauma [7], without drawing direct pathophysiological considerations. We are not aware of cases of post-operative hemicrania continua being published. We report a series of three patients who developed a continuous hemicranial headache in close temporal relation to cranial surgery and discuss clinical and pathophysiological implications.

## Findings

### Case 1

This 44-year old male was seen in our headache outpatient clinic because of a constant left-sided pain. The pain started three months after excision of a left-sided vestibular Schwannoma. He did not report any accompanying cranial autonomic symptoms. He did not report migrainous symptoms, as photophobia, phonophobia or nausea. However, the pain was described dull and with moderate intensity, worse with movement or sneezing. He was treated with diclofenac, ibuprofen and paracetamol in adequate doses, with no effect on the headache. On indomethacin 75 mg twice daily he had a total relief of the pain. When he once forgot to take the tablet, the pain returned within a few hours. At four months follow up, the headache was still fully controlled on indomethacin.

Other than his presenting problem, he had episodic migraine over years, which was treated with magnesium and riboflavin. He still reported irregular migraine attacks, which clearly differed from the current headache condition. He took mirtazapine for a sleeping problem. A recent MRI of the head did not show regrowth of the tumour or any other pathology, the neurological examination was normal.

### Case 2

A 26-year old man was treated for refractory temporal lobe epilepsy with a left-sided selective amygdalo-hippocampectomy with extirpation of temporo-mesial dysplasia. He had a skull bone infection with osteomyelitis, which had to be revised with two more operations. From the 2^nd^ operation he was seizure free on lamotrigine 400 mg/d. However, a few days after last intervention he developed an ongoing left-sided headache with moderate to severe pain. He also reported accompanying ptosis and lacrimation of the left eye with pain exacerbations. Apart of a residual concentric visual deficit on the left and the left-temporal skull defect with hypaesthesia, he had a normal neurological examination. He tried paracetamol and metamizol with no effect on the pain. After consultation treatment with indomethacin was started: 1^st^ week 75 mg, 2^nd^ week 150 mg and from 3^rd^ week 225 mg p.o. The pain level decreased immediately during the first week, resulting in a pain-free state on 75 mg three times daily. He had no past history of migraine or any other neurological disease.

### Case 3

A 51-year old lady had resection of a right-sided vestibular Schwannoma. Nearly four months later she developed a continuous right-sided headache of moderate intensity. With exacerbations of the pain she reported ipsilateral lacrimation and facial hot flushes. In addition she had irregular migraine attacks 2-3 times per month, which she reported differently, occurring bilaterally and accompanied by nausea and photophobia. Metamizol, paracetamol and triptans had no effect on the pain. She had a persistent ipsilateral vestibulocochlear dysfunction and a local hypaesthesia on the right forehead. The remaining neurological examination was normal. She was pain free on 150 mg of indomethacin for a few weeks. However, she did not tolerate the gastro-intestinal side effects in the long term and was put on gabapentin, which only provided partial relief. Other than that she had no past history of any other neurological disease.

## Discussion

The differential diagnosis of a continuous half-sided headache is not broad and includes hemicrania continua, unilateral chronic migraine, new daily persistent headache (NDPH), and paroxysmal hemicrania (PH) with interictal background pain [8]. HC can be differentiated from chronic migraine and NDPH by the positive response to indomethacin, whereas HC typically has less prominent cranial autonomic features, long lasting and less frequent painful exacerbations than PH [8]. HC is a rare disorder. The occurrence of HC-like headache of such a homogenous phenotype after craniotomy therefore seems to be more than pure coincidence.

While each of the three patients suffered from continuous hemicranial pain, only two had accompanying unilateral cranial autonomic symptoms (see Table 1). All patients fulfilled criterion D of the ICHD-3 beta for Hemicrania continua [3], which is the response to therapeutic doses of indomethacin. The indomethacin-effect might not be specific, in other words the strong NSAID might give relief to other pain syndromes, as reviewed by Trucco et al. [9]. However, the three reported cases did not have pain relief with other NSAIDs they had previously used. In addition, indomethacin-resistant unilateral headache has been reported in the literature [10]. Summ et al. showed that indomethacin inhibits nitric oxide induced vasodilation, nevertheless the exact mechanism in indomethacin-responsive headaches remains unclear [11].

**Table 1:** **The table shows the demographic details and the headache characteristics**

**n**	**Age, sex**	**Method of surgery**	**Time to onset after surgery**	**Headache type**	**Accompanying symptoms**	**Response to Indomethacin**	**Migraine**
1	44, m	left-sided vestibular schwannoma resection	3 months	left-sided continuous	none	total relief on 75 mg twice daily	+
2	26, m	left-sided selective amygdala-hypocampectomy	within few days	left-sided continuous	ipsilateral ptosis and tearing with exacerbations	total relief on 75 mg three times daily	-
3	51, f	right-sided vestibular schwannoma resection	4 months	right-sided continuous	ipsilateral tearing with exacerbation	total relief on 150 mg, not tolerated	+

Two of the patients reported in this series had a prior migraine history suggesting chronic migraine. However, the described headaches do not fulfil the diagnostic criteria for chronic migraine. As Cittadini and colleagues showed, HC might be more clinically variable than known so far, and its phenotype may show features of migraine or the Trigeminal Autonomic Cephalalgias (TACs) [2]. This has been taken into account when revising the TACs section of ICHD-3 beta.

A recent study of Schankin et al. analysed headache syndromes after acoustic neuroma surgery. Interestingly they found a severe type of tension-type headache and several with occipital neuralgia, but no similar half-sided headaches [12]. The patients in our series would not meet ICHD-3 beta criteria for post-craniotomy headache, as the area of maximal pain was greater than the area of the craniotomy, and two of them developed headache later than seven days after craniotomy. Although all three patients had different neurosurgical approaches: one fronto-temporal and two occipital, they all refer to the trigeminal-cervical pathway, first-ophthalmic trigeminal branch and greater occipital nerve. Taking this all together we suggest that the patients presented share an underlying pathophysiology, comparable to that of hemicrania continua. One way to consider this group is the notion that any cranial trauma: fracture, surgery, infection, can produce headache in biologically susceptible individuals- post-traumatic headache. Here one might think of the trauma as the pathophysiological trigger. The expression of post-traumatic headache may depend on the underlying biotype of the patient - in our cases the biotype of hemicrania continua. Further prospective studies of post-operative headaches are needed.
